# HPV Vaccination Among Students of Health-Related Disciplines: Knowledge, Intentions, Attitudes, and Barriers

**DOI:** 10.3390/vaccines14100907

**Published:** 2026-10-18

**Authors:** Sylwia Kałucka, Zuzanna Górczak, Izabela Grzegorczyk-Karolak

**Affiliations:** 1 Department of Coordinated Care, Medical University of Lodz, 90-251 Lodz, Poland; sylwia.kalucka@umed.lodz.pl; 2 Student Research Circle at the Department of Coordinated Care, Medical University of Lodz, 90-251 Lodz, Poland; zuzanna.gorczak@student.umed.lodz.pl; 3 Department of Biology and Pharmaceutical Botany, Medical University of Lodz, 90-151 Lodz, Poland

**Keywords:** human papillomavirus, HPV vaccination, vaccination uptake, vaccine acceptance, vaccine knowledge, vaccination barriers, health sciences students

## Abstract

**Background:** Although HPV vaccination is an effective cancer-prevention strategy, vaccination uptake among young adults remains suboptimal in many countries despite generally positive attitudes towards vaccination. The aim of this study was to assess HPV-related knowledge, vaccination uptake and intentions, awareness of the national vaccination programme, and attitudes towards vaccination among university students in health-related degree programmes. **Methods:** A cross-sectional questionnaire-based study was conducted among 428 students of Public Health, Dietetics, and Physiotherapy. **Results:** Basic HPV knowledge was high: 94.9% correctly identified the meaning of HPV and 95.3% identified sexual transmission as the main route of transmission. However, recognition of specific HPV risk factors was heterogeneous. Overall, 32.5% of students had completed the vaccination schedule or were in the process of vaccination, while 57.5% were unvaccinated but willing to vaccinate. The initiation of HPV vaccination was significantly associated with sex (*χ*^2^(1) = 18.739, *p* < 0.001; OR = 2.77, 95% CI: 1.73–4.44), but not with field of study (*p* = 0.370). Awareness of the national free HPV vaccination programme was high (91.1%). Most respondents recognised the public-health importance of HPV vaccination and supported its promotion in schools. General support for vaccination was also high (93.1%). **Conclusions:** This study identified a substantial gap between HPV vaccination uptake and vaccination intention among university students taking health-related subjects. Despite high basic HPV awareness and generally favourable attitudes towards vaccination, some gaps remained in specific vaccination-related knowledge. Interventions should therefore combine targeted education with measures addressing barriers to vaccination, with the aim of translating vaccination intentions into higher vaccine uptake.

## 1. Introduction

Few interventions in cancer prevention present as clear an opportunity as human papillomavirus (HPV) vaccination. Persistent infection with high-risk HPV types could be a cause of cervical cancer and contributes to a substantial proportion of anogenital and oropharyngeal malignancies in both women and men [1,2]. Vaccination can prevent infection by HPV and subsequent disease years or even decades before its clinical manifestation. Indeed, national HPV vaccination programmes worldwide have achieved substantial reductions in HPV infection and cervical pre-cancer. As such, vaccination should be regarded as a central component of contemporary cancer prevention strategies [3,4,5].

Nevertheless, cervical cancer remains a major global public health problem, with approximately 604,000 new cases and 280,000 deaths estimated worldwide in 2024. The World Health Organization (WHO) has defined cervical cancer elimination as a public health goal and established the 90–70–90 targets, with at least 90% of girls recommended to be fully vaccinated against HPV by 15 years of age [6]. However, despite the substantial expansion of HPV vaccination programmes, global vaccination coverage remains insufficient. In 2025, despite HPV vaccination being included in national immunisation programmes and services in 162 countries, global coverage with the first dose of HPV vaccine among girls was estimated at 33% [7].

The discrepancy between the demonstrated effectiveness of HPV vaccination and its population-level uptake highlights an important implementation challenge. HPV vaccination is primarily a preventive intervention, and its benefits are often temporally distant from the decision to vaccinate. Consequently, vaccination behaviour may be shaped by multiple interacting factors, including knowledge, perceived risk, confidence in vaccine safety and effectiveness, social norms, healthcare-provider recommendation, affordability, accessibility, and the organisation of vaccination services [8,9,10,11].

University students in many countries represent an important population in this context. Young adults who have not received HPV vaccination during adolescence may subsequently need to make an individual decision about vaccination, while access to publicly funded programmes may differ substantially from that available to younger adolescents. A recent systematic review and meta-analysis including 56 studies and 184,351 university students from four continents reported that approximately half of students were willing to receive HPV vaccination, whereas only 10.4% had received at least one dose. The review also identified important gaps in knowledge concerning vaccination timing and HPV-related disease [12]. These findings indicate that knowledge, willingness, and actual vaccination uptake represent related but distinct dimensions of HPV vaccination behaviour.

Healthcare professionals play an important role in this process because they constitute an important source of vaccination information and recommendation [13]. However, evidence indicates substantial variation in HPV vaccine recommendation practices among healthcare workers. A systematic review and meta-analysis of 73 studies identified concerns regarding vaccine safety and efficacy, cost, parental concerns, and systemic barriers among factors associated with healthcare workers’ HPV vaccination recommendation practices [14].

Hence, the students preparing for healthcare-related professions represent a particularly suitable population for investigation. They occupy a transitional position between healthcare recipients and future health communicators. Their own vaccination behaviour may provide an insight into the barriers faced by young adults, while their knowledge and attitudes may also be relevant to their future capacity to participate in health promotion and preventive care. Importantly, vaccination communication is not restricted to physicians. Public health specialists, physiotherapists, dietitians, nurses, and other health professionals may encounter patients in settings in which preventive health behaviours are discussed.

The information environment in which these decisions are made has also become increasingly complex. Young adults frequently obtain health information from digital and social media platforms, where evidence-based information coexists with misleading claims. HPV vaccination may be particularly susceptible to misinformation because discussions may involve sexuality, reproductive health, cancer, and vaccine safety. Recent research examining HPV vaccine-related social media content demonstrated pervasive misinformation and substantial variation in misinformation themes between platforms [15].

The Polish setting provides an informative context in which to examine these issues. A nationwide fully funded public HPV vaccination programme was introduced in Poland in June 2023, initially targeting girls and boys aged 12–13 years, with vaccination offered on a voluntary basis [16]. From September 2024, the programme was expanded to children from nine years of age until completion of 14 years of age, with vaccination available through primary healthcare and, under defined arrangements, in schools. Thus, many current university students reached the recommended age for HPV vaccination before the establishment of the current nationwide publicly funded programme. Their vaccination status therefore needs to be interpreted within a different policy and access environment from that experienced by younger cohorts.

This distinction is particularly important when interpreting low HPV vaccination uptake. Non-vaccination does not necessarily indicate rejection of vaccination. Individuals may accept vaccination in principle but remain unvaccinated because of cost, lack of recommendation, uncertainty regarding eligibility, limited access, or the absence of an organised opportunity to receive the vaccine. Distinguishing between these possibilities is important for designing interventions that address both vaccine confidence and practical access.

The present study assessed HPV-related knowledge, vaccination status, perceived financial barriers, awareness of the national HPV vaccination programme, and general attitudes towards vaccination among students from three health-related disciplines: Public Health, Dietetics, and Physiotherapy. By including students from diverse healthcare-oriented fields rather than focusing on medical students, the study aimed to provide a broader perspective on HPV vaccination-related knowledge and attitudes among future health professionals. Particular attention was paid to differences between the study groups in awareness of the national vaccination programme and sources of information about HPV vaccination. The study also examined the discrepancy between HPV vaccination initiation and willingness to vaccinate among respondents who had not yet received any HPV vaccine dose. This approach was intended to distinguish descriptive patterns of vaccination behaviour from broader interpretations of vaccine hesitancy and to identify potentially relevant areas for educational and health-system interventions.

## 2. Materials and Methods

### 2.1. Study Design and Participants

A cross-sectional questionnaire-based study was conducted among students of the Faculty of Health Sciences at the Medical University of Lodz, Poland. The study included students enrolled in three health-related degree programmes: Public Health, Dietetics, and Physiotherapy. Data were collected between October 2024 and January 2025. The target population comprised 1390 students enrolled in the three programmes, all of whom were invited to participate. Recruitment was based on voluntary, non-probability sampling. An electronic version of the questionnaire was distributed via an online survey platform. A total of 428 completed questionnaires were obtained, corresponding to an approximate response rate of 30.8% of the target population.

### 2.2. Ethical Considerations

This study was conducted in accordance with the principles of the Declaration of Helsinki. The study protocol was reviewed and approved by the Bioethics Committee of the Medical University of Lodz, Poland (RNN/207/23/KB, 11 July 2023).

Participation was voluntary and anonymous. Before completing the questionnaire, participants were informed online about the purpose of the study, the voluntary nature of participation, the anonymous character of data collection, and the use of the collected information for scientific purposes. Participants were also informed that completion of the questionnaire was considered to constitute informed consent to participate in the study. No personally identifiable information was collected.

### 2.3. Questionnaire Development and Data Collection

Data were collected using an anonymous, investigator-developed questionnaire designed specifically for the present study. Before the main survey, the questionnaire underwent pilot testing and content refinement. A preliminary version was administered to a small group of students to assess the clarity, comprehensibility, and relevance of the individual questions. Participants’ feedback was subsequently discussed among the investigators, and selected items were clarified, modified, or reformulated before the final version was administered.

The final questionnaire comprised 33 items covering six thematic domains: (I) sociodemographic and educational characteristics, including age, field and level of study, sex, place of origin, sexual initiation, and recent use of gynaecological or urological healthcare services; (II) HPV vaccination status, access, and attitudes, including current vaccination status, sources of vaccination recommendations, perceived financial barriers, and motivations for HPV vaccination; (III) basic knowledge of HPV and HPV vaccination, including knowledge of the meaning of the HPV abbreviation, the main route of HPV transmission, selected risk factors for HPV infection in women and men, and general awareness of age-related recommendations for HPV vaccination; (IV) awareness of and attitudes towards the national free HPV vaccination programme, including awareness of the programme, knowledge of the age eligibility criteria, perceptions of programme promotion, and the willingness to promote HPV vaccination and provide HPV-related education in schools; (V) previous experience with recommended (non-mandatory) vaccination, including COVID-19 and influenza vaccination, vaccination-related fear or concerns, and reasons for such concerns; and (VI) general attitudes towards vaccination, including support for vaccination in general and childhood vaccination, attitudes towards vaccination during pregnancy, willingness to promote recommended vaccinations as a healthcare professional, and seeking information about recommended vaccinations before travel to destinations outside Europe.

The questionnaire included single-choice, multiple-choice, and open-ended questions, as well as individual items rated on a five-point Likert-type agreement scale ranging from “Strongly agree” to “Strongly disagree.” Selected questions, including those concerning HPV risk factors and motivations for HPV vaccination, allowed respondents to select multiple answers. Some questions concerning previous vaccination experiences and vaccination-related concerns were presented conditionally, depending on respondents’ previous vaccination status or reported concerns. Regarding the question, “Who recommended that you get vaccinated against HPV?”, although the accompanying instruction indicated that the question was intended for vaccinated respondents, respondents across HPV vaccination-status categories answered it. Therefore, responses to this question were analysed using the entire study sample as the denominator.

The complete English-language version of the questionnaire is provided in the Supplementary Material (Figure S1).

### 2.4. Outcome Measures and Data Recoding

The main outcome was HPV vaccination initiation, defined as receipt of at least one dose of HPV vaccine. For descriptive purposes, HPV vaccination status was divided into four categories: participants who had completed the vaccination schedule, those who were still in the process of vaccination, those who had not been vaccinated but were willing to receive the vaccine, and those who had not been vaccinated and were unwilling to receive it. For statistical analyses, vaccination status was additionally dichotomized into two groups: participants who had initiated HPV vaccination (i.e., those who had received at least one dose, regardless of whether they had completed the vaccination schedule) and those who had not received any HPV vaccine dose, irrespective of their intention to receive the vaccine.

Additional variables included individual HPV knowledge items, knowledge about HPV vaccination and the national publicly funded HPV vaccination programme, perceived financial barriers, sources of vaccination recommendations, attitudes towards HPV vaccination and vaccination in general, willingness to promote HPV vaccination and provide HPV-related education in schools, and previous COVID-19 and influenza vaccination.

Variables with binary response options in the original questionnaire were analysed without recoding. Where required for inferential analyses, variables with multiple response categories were recoded into binary categories according to predefined analytical criteria by combining conceptually similar categories. These included COVID-19 vaccination (≥1 dose vs. no vaccination), influenza vaccination (vaccinated vs. not vaccinated), knowledge of programme eligibility (correct response: “girls and boys aged 9–14 years” vs. other responses, including “I do not know”), and selected Likert-type vaccination attitude items (strongly agree/agree vs. no opinion/disagree/strongly disagree).

For multiple-response questions, each response option was analysed as a separate categorical variable. Therefore, percentages for individual response options were not expected to sum to 100%.

### 2.5. Statistical Analyses

Statistical analyses were performed using Statistica 13.1 PL (StatSoft, Tulsa, OK, USA). Given the cross-sectional and exploratory design, analyses were primarily descriptive and bivariate. Categorical variables are summarised as frequencies and percentages [*n* (%)].

Associations between field of study (Public Health, Dietetics, and Physiotherapy) and selected vaccination-related variables were assessed using Pearson’s chi-square (*χ*^2^) test. When the expected cell frequencies did not meet the assumptions of Pearson’s *χ*^2^ test, an exact test was used. In particular, differences in influenza vaccination status across the three degree programmes were assessed using the Fisher–Freeman–Halton exact test for a 2 × 3 contingency table because of sparse cell frequencies.

Associations between sex and selected vaccination-related variables were assessed using Pearson’s *χ*^2^ test for 2 × 2 contingency tables or Fisher’s exact test when expected cell frequencies were small. For statistically significant associations, odds ratios (ORs) with 95% confidence intervals (CIs) were calculated to quantify the strength and direction of the association between sex and binary vaccination-related outcomes.

For statistically significant omnibus associations involving field of study, three pairwise comparisons between degree programmes were subsequently performed using Pearson’s *χ*^2^ test for 2 × 2 tables; Fisher’s exact test was used when the expected frequencies were small. *p*-values were adjusted using the Holm procedure.

## 3. Results

### 3.1. Study Population

This study included 428 students: 174 from Public Health, 171 from Dietetics, and 83 from Physiotherapy (Table 1). Women comprised 64.7% of the sample, with a similar sex distribution across the three fields of study. The mean age of participants was approximately 21 years. Most respondents reported having initiated sexual activity (90.4%), whereas 55.4% had visited a gynaecologist or urologist within the previous two years. The proportion of participants reporting a recent gynaecological or urological consultation differed by sex; approximately 30% of women had not consulted a gynaecologist within the previous two years, compared with approximately 65–80% of men who had not consulted a urologist. This pattern is consistent with the young age of the study population and the differences in the routine need for specialised preventive care between young women and men.

### 3.2. HPV Knowledge

Basic knowledge of HPV was high among the study participants (Table 2). Overall, 94.9% of respondents correctly identified the meaning of the abbreviation HPV, and 95.3% correctly identified sexual transmission as the main route of HPV transmission.

Knowledge of HPV risk factors was more heterogeneous. The factors included in the questionnaire were selected based on established epidemiological and clinical associations with HPV infection or HPV-related disease [17,18], but their recognition varied substantially. Recognition of specific HPV risk factors was more heterogeneous. The most frequently identified risk factor for HPV infection in both women and men was interaction with multiple sexual partners, as reported by 59.1% and 62.6% of respondents, respectively. Immunosuppression was also frequently identified as a risk factor, whereas early sexual activity, smoking, and other listed risk factors were identified less consistently.

Responses to the question concerning the age-related recommendations for HPV vaccination were highly heterogeneous. Most respondents (87.1%) selected the option “Anyone, regardless of age”, whereas 6.1% selected “People from 9 years of age”. The remaining respondents selected other options or indicated that they did not know.

### 3.3. HPV Vaccination Uptake, Intentions and Attitudes

Overall, 32.5% of respondents had initiated HPV vaccination, including 22.7% who had completed the vaccination schedule and 9.8% who were still in the process of vaccination (Table 3). Among those who had not received any HPV vaccine dose, 57.5% declared willingness to be vaccinated. In contrast, 10.0% were unvaccinated and declared no willingness to receive HPV vaccination. Thus, although the level of HPV vaccination initiation was relatively low, a large proportion of non-initiators expressed an intention to receive the vaccine in the future.

The prevalence of HPV vaccination initiation did not differ significantly between the three fields of study: Public Health, Dietetics, and Physiotherapy (Pearson’s *χ*^2^(2) = 1.989, *p* = 0.370) (Table S1). In contrast, vaccination initiation was significantly associated with sex (*χ*^2^(1) = 18.739, *p* < 0.001; OR = 2.77, 95% CI: 1.73–4.43) (Table S2): the female participants exhibited a higher likelihood of having initiated HPV vaccination.

The most frequently reported reasons for HPV vaccination were protection of one’s own health (80.6%) and protection of a sexual partner from HPV infection (72.2%) (Table 3). The prevention of specific HPV-related cancers was selected less frequently.

Financial considerations represented a potential barrier for a substantial minority of respondents. Approximately half (50.7%) reported that the cost of HPV vaccination would not be a problem, while 33.4% considered the cost a difficulty but would nevertheless proceed with vaccination. For 11.9% of respondents, the financial burden had led to or would lead to abandonment of vaccination, whereas 4.0% indicated that cost was irrelevant because they would not vaccinate regardless (Table 3).

Academic teachers were the most frequently reported identifiable source of HPV vaccination recommendations (34.3%), followed by parents (17.1%) (Table 3). Recommendations from primary care physicians (2.6%) and specialist physicians (1.2%) were uncommon, while 43.2% of respondents reported that nobody had recommended HPV vaccination.

### 3.4. Awareness of and Attitudes Towards the National HPV Vaccination Programme

Awareness of the national publicly funded HPV vaccination programme was high overall, with 91.1% of respondents reporting that they were aware of its existence (Table 4). The proportion of students aware of the programme differed significantly across fields of study (Pearson’s *χ*^2^(2) = 15.281, *p* < 0.001), with the highest awareness observed among Dietetics students (97.7%), compared with 87.4% of Public Health students and 85.5% of Physiotherapy students (Table S1).

Despite the high level of awareness, perceptions of the sufficiency of programme promotion were more moderate. Overall, 60.5% of respondents considered the promotion of the national HPV vaccination programme sufficient (Table 4). This perception also varied across fields of study, with the highest proportion of students reporting sufficient promotion among Dietetics students (66.1%) and the lowest among Physiotherapy students (49.4%).

Knowledge of programme eligibility was also significantly associated with field of study (Pearson’s *χ*^2^(2) = 8.743, *p* = 0.013), with the correct response: “girls and boys aged 9–14 years” selected by 73.6% of respondents overall. Dietetics students demonstrated the highest level of correct knowledge (80.7%), compared with 66.7% among Public Health and 73.5% among Physiotherapy students; a significant pairwise difference was observed only between Public Health and Dietetics (*p* = 0.009).

The importance of HPV vaccination for public health was widely recognised: 90.0% of respondents considered HPV vaccination important for public health (Table 4).

Respondents also expressed substantial support for involving final-year students of health-related disciplines in promoting HPV vaccination within the school community. Overall, 81.3% believed that final-year students should promote HPV vaccination in schools. This proportion was highest among Dietetics students (85.4%) and Public Health students (83.3%), and lower among Physiotherapy students (68.7%). The association with field of study was statistically significant (Table S1), with significant differences between Public Health and Physiotherapy (*p* = 0.014; OR = 2.28, 95% CI: 1.24–4.20) and between Dietetics and Physiotherapy (*p* = 0.005; OR = 2.66, 95% CI: 1.42–4.99).

Willingness to personally deliver HPV education in schools was somewhat lower but remained substantial, with 60.0% of respondents reporting a willingness to do so (Table 4). This measure also differed significantly by field of study (Pearson’s *χ*^2^(2) = 6.217, *p* = 0.045), although no significant pairwise differences remained after Holm adjustment (Table S1).

### 3.5. Previous Experience with Other Recommended (Non-Mandatory) Vaccinations

Previous experience with COVID-19 vaccination was common in the study population (Table 5). Overall, 87.1% of respondents had received at least one COVID-19 vaccine dose, including 31.5% who had received three doses and 2.1% who had received more than three doses. The COVID-19 vaccination status did not differ significantly between the three fields of study (Pearson’s *χ*^2^(2) = 0.162, *p* = 0.922) or between women and men (*χ*^2^(1) = 0.180, *p* = 0.671) (Tables S1 and S2).

Among respondents who had received at least one COVID-19 vaccine dose, 36.7% reported having experienced fear before their first vaccination. The most commonly reported reason was fear of adverse effects (62.8%), followed by lack of trust in COVID-19 vaccines (32.8%) (Table 5). Fear related to the first COVID-19 vaccination did not appear to reflect a generalised lack of trust in vaccines, as none of the respondents selected lack of trust in vaccines in general as a reason for their concern.

In contrast to COVID-19 vaccination, influenza vaccination was uncommon. Only 3.0% of respondents reported having received an influenza vaccine within the previous three years, corresponding to 13 individuals in the entire study population. Vaccination was reported by 4.6% of Public Health students, none of the Dietetics students, and 6.0% of Physiotherapy students (Table 5).

An exact test indicated an association between field of study and influenza vaccination status (Fisher–Freeman–Halton exact test, *p* = 0.003), with significant pairwise comparisons between Dietetics and Public Health (*p* = 0.014) and between Dietetics and Physiotherapy (*p* = 0.010) (Table S1). However, these findings should be interpreted with caution because of the very small number of vaccinated respondents and the absence of vaccinated participants in the Dietetics group. No significant association was observed between sex and influenza vaccination status (Fisher’s exact test, *p* = 0.557). Given the low number of influenza vaccine recipients, this analysis also provides limited statistical power and precision for detecting associations with sex.

### 3.6. General Attitudes Towards Vaccination

Overall, the findings indicate a consistently pro-vaccination orientation across this study population (Table 6). General support for vaccination was expressed by 92.5% of participants, including 68.2% who strongly agreed with the statement. Support was highest among Physiotherapy students (96.3%), followed by Dietetics (95.3%) and Public Health students (88.0%). The distribution of responses differed significantly by field of study (Pearson’s *χ*^2^(2) = 9.030, *p* = 0.011), with a significant pairwise difference between Dietetics and Public Health (*p* = 0.040) (Table S1).

Support for childhood vaccination was even more pronounced, with 94.4% of respondents agreeing or strongly agreeing that children should be vaccinated (Table 6). No significant association with field of study was observed (*χ*^2^(2) = 3.951, *p* = 0.139).

In contrast, attitudes towards vaccination during pregnancy were more heterogeneous (Table 6). Although 63.3% of respondents agreed or strongly agreed that pregnant women should be vaccinated, 31.80% expressed no opinion and 4.9% disagreed or strongly disagreed. The distribution did not differ significantly between fields of study (Table S1).

Support for vaccination promotion among healthcare professionals was also high, with 83.1% of respondents agreeing or strongly agreeing that they would promote recommended non-mandatory vaccinations among healthcare professionals (Table 6). No significant differences were observed between fields of study (Table S1). Similarly, 94.1% of respondents reported an interest in recommended vaccinations before travelling to exotic destinations, such as countries in Asia or Africa, with no significant association with field of study (Table 6 and Table S1).

## 4. Discussion

The present study demonstrates an important distinction between general vaccine acceptance, HPV awareness, vaccination-specific knowledge, and actual HPV vaccination uptake among students preparing for health-related professions. Although basic awareness of HPV was high and attitudes towards vaccination were generally favourable, only 32.5% of respondents had initiated HPV vaccination. Among those who had not received any dose, 57.5% expressed an intention to receive HPV vaccination. This pattern suggests that the principal challenge in this population may not be widespread rejection of vaccination, but rather a gap between favourable attitudes and their translation into vaccination behaviour. This interpretation is consistent with a recent systematic review and meta-analysis of 56 studies including more than 184,000 university students, which estimated willingness to receive HPV at 52.9% but actual uptake at only 10.4% [12]. Importantly, the individual studies included in that review also demonstrate that vaccination behaviour varies considerably according to age, sex, field of study, access, and healthcare context. Thus, non-vaccination should not automatically be equated with vaccine hesitancy. In the present study, most unvaccinated respondents expressed a willingness to receive the vaccine, with only 10.0% declining. This suggests that although a degree of HPV-specific reluctance was present, it did not account for the non-vaccination status of the entire group. Furthermore, the fact that the attitude towards vaccination was generally favourable, with no respondents expressing opposition to vaccination in general, suggests an additional indication of overall vaccine acceptance, albeit not an equivalent one.

The high proportion of respondents correctly identifying HPV and its sexual transmission indicates a high level of basic HPV awareness in this health-related student population; however, this awareness was not accompanied by equally comprehensive knowledge of HPV vaccination. Responses to the question concerning age-related recommendations for HPV vaccination were highly heterogeneous: most respondents (87.1%) selected “Anyone, regardless of age”, whereas only 6.1% selected “People from 9 years of age”. When interpreting this finding, it is worth considering the separate question on eligibility for the national vaccination programme, in which 73.6% of respondents correctly identified girls and boys aged 9–14 years as eligible. Hence, as a group, the participants exhibited more complete knowledge of the specific eligibility criteria for the national programme than of the broader age-related recommendations for HPV vaccination. It should be noted that these findings do not necessarily indicate that the same respondents were familiar with the programme criteria but lacked knowledge of general vaccination recommendations: no comparison was performed of the individual response patterns between the two questions.

The distinction between these two aspects is important in light of the WHO recommendations. According to the 2022 WHO position paper, the primary target group for HPV vaccination is girls aged 9–14 years, with vaccination ideally provided before potential exposure to HPV. While the recommendations address secondary target groups such as older girls, women and boys, its implementation depends on feasibility and affordability and should not compromise vaccination of the primary target group [19]. In Poland, eligibility for the publicly funded national programme during the study period was restricted to girls and boys aged 9 to 14 years [16]. Thus, eligibility for the national programme constituted a specific funding criterion not the full scope of recommendations concerning HPV vaccination. The wording and response options of the first questionnaire item did not adequately distinguish between the primary target group for routine vaccination and older individuals who had not previously been vaccinated, and they did not provide a definitive assessment of knowledge of specific age-related recommendations. Consequently, the popularity of “Anyone, regardless of age” as a response should not be taken as conclusive evidence that respondents considered vaccination appropriate at literally any age.

Nevertheless, the results point to the importance of educational interventions that go beyond general HPV awareness and address specific, decision-relevant information about vaccination, particularly among future healthcare professionals who may become sources of information and recommendations for patients and the wider community [20]. Similar discrepancies between basic HPV awareness and more specific vaccination-related knowledge have been reported among university students in China and other countries [21,22]. A recent study among health-science students in Turkey also identified gaps in knowledge concerning HPV, HPV testing, vaccination, and vaccine accessibility [23,24].

The relatively low level of HPV vaccination initiation in the present cohort should also be interpreted in the context of the vaccination opportunities available to this generation. Most participants were approximately 21 years old at the time of the study and had passed through the primary age range for HPV vaccination before the introduction of the nationwide publicly funded programme in Poland. Consequently, unlike younger cohorts currently eligible for publicly funded vaccination, their opportunities to receive the vaccine during adolescence depended largely on parental or individual initiative, healthcare-provider recommendation, and the ability to cover the cost. The financial responses provide further insight into this context. Although 50.7% of respondents reported that the cost of HPV vaccination was not a problem, 33.4% considered it a financial difficulty but nevertheless regarded vaccination as worthwhile, while 11.9% selected the option indicating that vaccination had been abandoned because of its financial burden. These findings identify perceived financial constraints as one of the factors potentially limiting HPV vaccination, although their relationship with vaccination initiation was not analysed in this study. Similar concerns about cost have been identified in other studies of HPV vaccination among young adults and healthcare students [14,22,25]. Consequently, these insights could be vital when interpreting the intention–uptake gap in a cohort whose access to vaccination during adolescence differed from that of younger generations. University-based vaccination initiatives could help improve access among young adults; however, their effectiveness may depend on whether vaccination is accompanied by financial support or reimbursement.

The contemporary information environment provides an additional context for interpreting HPV vaccination attitudes. In the present study, academic teachers were among the more frequently reported sources of vaccination-related information, whereas recommendations from primary care physicians and medical specialists were reported relatively infrequently. At the same time, no external recommendation and self-directed information seeking were frequently cited. Although independent information seeking may reflect active engagement with health-related decisions, reliance on self-directed searches without adequate professional guidance may also increase exposure to inconsistent or inaccurate information. This is particularly relevant in the context of HPV vaccination, where information is readily available through social media and other online platforms.

Social media constitute an important source of health information for young people, but they also provide an environment in which inaccurate information can circulate widely. Boatman et al. [15] analysed 2996 social-media comments concerning HPV vaccination across three platforms and found pervasive misinformation, with recurring themes involving adverse reactions, the perceived unnecessary nature of vaccination, conspiracy theories, and mistrust of authorities. Such misinformation may be particularly problematic for HPV vaccination because the topic intersects with sexuality, reproductive health, and cancer prevention; claims concerning infertility or other reproductive consequences may therefore have strong emotional salience. In this context, the combination of predominantly self-directed information seeking, incomplete vaccination-specific knowledge, and the relatively limited role of healthcare professionals as reported information sources highlights the importance of accessible, evidence-based and professionally endorsed information.

The limited role of healthcare professionals is particularly noteworthy because a clear recommendation from a healthcare provider is consistently identified as an important facilitator of HPV vaccine uptake. In the present study, more than 40% of respondents reported that nobody had recommended HPV vaccination. This is noteworthy given the important role of healthcare workers (HCWs) in providing vaccine information and influencing vaccination decisions. However, HPV vaccine recommendation practices vary substantially across regions, with much higher rates reported among HCWs in Europe than in the Eastern Mediterranean region [15]. Concerns about vaccine efficacy and safety may reduce HCWs’ willingness to recommend HPV vaccination, while higher HPV-related knowledge was associated with a greater likelihood of recommending HPV vaccination among healthcare professionals [13,14,26]. Importantly, insufficient knowledge or vaccine hesitancy among HCWs may also limit their ability to address patients’ and parents’ concerns and provide appropriate, evidence-based reassurance. This is particularly relevant given that concerns and uncertainty about vaccine safety and effectiveness remain important barriers to vaccination in the general population [27]. Although the present study cannot determine whether the absence of a professional recommendation contributed to non-vaccination, the low frequency of reported recommendations suggests an underused opportunity for proactive communication, particularly among young adults who passed through adolescence without receiving the vaccine.

The contrast between HPV vaccination and previous vaccination against COVID-19 and influenza provides another perspective on the relationship between attitudes and behaviour. COVID-19 vaccination was common in the present cohort, with 87.1% reporting at least one dose, whereas only 3.0% reported influenza vaccination during the preceding three years. This pattern suggests that vaccination behaviour may be strongly context-dependent. COVID-19 vaccination occurred during an acute and highly visible public-health emergency accompanied by intensive communication, institutional mobilisation, and a high perceived immediate risk. Seasonal influenza, in contrast, may be perceived as a less immediate threat by young adults. A Polish study among medical students provides an illustrative example: influenza vaccination coverage increased markedly in 2020 in the context of a university campaign providing free vaccination and heightened concern during the COVID-19 pandemic [28]. These observations suggest that general vaccine acceptance does not necessarily predict uptake when perceived urgency, accessibility, recommendation intensity, and opportunity differ between vaccines. The same principle may apply to HPV vaccination, for which the health consequences are long-term and the optimal vaccination window occurs years before most HPV-related disease becomes clinically apparent.

Differences between the three fields of study provide an additional and somewhat unexpected finding. Dietetics students demonstrated the highest awareness of the national HPV vaccination programme and the highest proportion correctly identifying programme eligibility. They also showed the most favourable general attitudes towards vaccination. Public Health students, despite their explicit professional orientation towards population health, prevention, and health promotion, did not consistently demonstrate the highest levels of awareness or general vaccine support. This finding should not be interpreted as evidence that one educational programme provides superior preparation, because the study did not assess curriculum content, previous exposure to vaccination education, or other potentially relevant factors. Nevertheless, it raises an important question concerning the extent to which vaccination and health communication are integrated across health-related curricula.

Support for HPV vaccination promotion by final-year students within the school community was high across all three fields of study, although willingness to personally provide HPV education was lower. This distinction may reflect differences in the willingness of the participants to assume an active role in HPV vaccination promotion and their perceived preparedness to undertake such activities, rather than negative attitudes towards HPV vaccination itself. This is particularly relevant given the documented gaps in HPV and vaccine knowledge among university students and the persistently low vaccination uptake reported across studies [12]. For future healthcare professionals, competence in vaccine communication should therefore complement factual knowledge. Training could include practical approaches to explaining vaccine eligibility and benefits, addressing questions concerning safety and adverse effects, correcting misconceptions, and directing individuals towards reliable information. Recent intervention-based evidence suggests that structured peer-education approaches can improve HPV-related knowledge and awareness among health-science students [29], while a randomised controlled trial showed that an HPV-focused educational intervention delivered by trained students from a health-related discipline was associated with higher HPV vaccination initiation among adolescents [30].

## 5. Strengths and Limitations

This study has several strengths. First, it included students from three health-related disciplines—Public Health, Dietetics, and Physiotherapy—rather than focusing exclusively on medical students, thereby providing a broader perspective on future healthcare professionals. Second, the questionnaire addressed several complementary aspects of HPV prevention, including HPV knowledge, vaccination uptake and intentions, financial barriers, awareness of the national vaccination programme, and attitudes towards vaccination. Third, the study was conducted during a period of substantial development of the national HPV vaccination programme, providing timely insight into vaccination-related knowledge and attitudes among young adults who largely reached adolescence before the current publicly funded programme was introduced.

Several limitations should be considered. The cross-sectional and exploratory design does not allow causal inferences. Associations between field of study and vaccination-related variables were examined using bivariate analyses without adjustment for potential confounders. Although the study groups were similar in terms of age and sex distribution, they differed with regard to the proportion of students at Bachelor’s and Master’s levels, which may have influenced some of the results. Therefore, these findings should be interpreted as unadjusted exploratory relationships. The participants were drawn as a voluntary, non-probability sample from a single university, which limits the generalizability of the findings. As no individual-level information was available regarding non-participants, non-response bias cannot be completely excluded. In addition, vaccination status and other health-related information were self-reported and were not verified against vaccination records; therefore, recall or reporting bias cannot be completely excluded. The questionnaire was developed by the investigators; while it underwent pilot testing and content refinement, it was not subjected to psychometric validation. As the instrument was designed as a structured epidemiological survey comprising heterogeneous items rather than as a psychometric scale, measures of internal consistency such as Cronbach’s alpha were not applicable to the questionnaire as a whole. Accordingly, the findings concerning knowledge and attitudes were interpreted at the level of individual survey items rather than as scores derived from a formally validated scale. Furthermore, although the questionnaire assessed willingness to receive HPV vaccination and perceived financial barriers, it did not comprehensively evaluate all potential reasons for non-vaccination or measure HPV vaccine hesitancy as a distinct construct. Therefore, the relative contribution of attitudinal and access-related factors to non-vaccination could not be determined. In addition, the question on who recommended HPV vaccination to the respondents was intended for vaccinated respondents but was analysed across the entire sample because respondents from all HPV vaccination-status categories provided answers. The original instruction may have influenced respondents’ interpretation of the question; therefore, the findings should be interpreted cautiously.

## 6. Conclusions

Students preparing for health-related professions demonstrate high basic awareness of HPV and generally favourable attitudes towards vaccination, but HPV vaccination initiation remained substantially lower in the group than vaccination intention. This gap highlights that favourable attitudes and vaccination intentions do not necessarily translate into vaccine uptake. The perceived financial barriers suggest that affordability remains a relevant concern for some students, while the historical lack of publicly funded HPV vaccination for the age cohort studied provides important context for interpreting the findings. More broadly, the differences observed between HPV, COVID-19, and influenza vaccination indicate that vaccine uptake may vary according to the specific context and accessibility of individual vaccines, rather than reflecting general attitudes towards vaccination alone. These findings highlight the importance of combining vaccination-specific information and effective communication with practical measures to facilitate access to HPV vaccination. Universities and healthcare institutions may have an important role in this regard, both by improving access to vaccination for young adults and by preparing future healthcare professionals to provide accurate, evidence-based information and support informed vaccination decisions. The multidisciplinary student population examined in this study is particularly relevant because these students represent both potential recipients of HPV vaccination and future contributors to health promotion and preventive care.

## Figures and Tables

**Table 1:** Demographic and selected characteristics of study participants by field of study.

Characteristic	Public Health (*n* = 174)	Dietetics (*n* = 171)	Physiotherapy (*n* = 83)	Total (*n* = 428)
**Level of study**				
Bachelor’s degree	103 (59.2%)	129 (75.4%)	59 (71.1%)	291 (68.0%)
Master’s degree	71 (40.8%)	42 (24.6%)	24 (28.9%)	137 (32.0%)
**Age** (mean ± SD)	21.4 ± 1.85	21.05 ± 1.83	20.7 ± 1.62	21.1 ± 1.81
**Sex**				
Female	114 (65.5%)	108 (63.2%)	55 (66.3%)	277 (64.7%)
Male	60 (34.5%)	63 (36.8%)	28 (33.7%)	151 (35.3%)
**Place of origin**				
City with >100,000 inhabitants	79 (45.4%)	75 (43.9%)	43 (51.8%)	197 (46.0%)
City with 20,000–100,000 inhabitants	49 (28.2%)	68 (39.8%)	16 (19.3%)	133 (31.1%)
City with <20,000 inhabitants	24 (13.8%)	14 (8.2%)	8 (9.6%)	46 (10.7%)
Village	22 (12.6%)	14 (8.2%)	16 (19.3%)	52 (12.1%)
**Sexual initiation**				
Yes	161 (92.5%)	152 (88.9%)	74 (89.2%)	387 (90.4%)
No	13 (7.5%)	19 (11.1%)	9 (10.8%)	41 (9.6%)
**Visit to a gynaecologist/urologist within the previous 2 years**				
Yes	91 (52.3%)	98 (57.3%)	48 (57.8%)	237 (55.4%)
No	83 (47.7%)	73 (42.7%)	35 (42.2%)	191 (44.6%)

Data are presented as *n* (%). Percentages were calculated within each field of study.

**Table 2:** Basic knowledge of HPV infection and vaccination.

Variable	Public Health (*n* = 174)	Dietetics (*n* = 171)	Physiotherapy (*n* = 83)	Total (*n* = 428)
**Knowledge of the HPV abbreviation**				
Yes	166 (95.4%)	161 (94.2%)	79 (95.2%)	406 (94.9%)
No	8 (4.6%)	10 (5.8%)	4 (4.8%)	22 (5.1%)
**Most common route of HPV transmission**				
Respiratory route	0 (0.0%)	0 (0.0%)	0 (0.0%)	0 (0.0%)
Foodborne route	0 (0.0%)	0 (0.0%)	0 (0.0%)	0 (0.0%)
Sexual route	161 (92.5%)	165 (96.5%)	82 (98.8%)	408 (95.3%)
Through the skin	13 (7.5%)	6 (3.5%)	1 (1.2%)	20 (4.7%)
Risk factors for HPV infection in women *				
Early sexual activity	84 (48.3%)	47 (27.5%)	30 (36.1%)	161 (37.6%)
Multiple sexual partners	140 (80.5%)	79 (46.2%)	34 (41.0%)	253 (59.1%)
Immunosuppression	100 (57.5%)	119 (69.6%)	55 (66.3%)	274 (64.0%)
Smoking	43 (24.7%)	97 (56.7%)	49 (59.0%)	189 (44.2%)
Infections such as chlamydia	22 (12.6%)	18 (10.5%)	12 (14.5%)	52 (12.1%)
Risk factors for HPV infection in men *				
Multiple sexual partners	144 (82.8%)	84 (49.1%)	40 (48.2%)	268 (62.6%)
Chronic urethritis and inflammation of the glans	34 (19.5%)	25 (14.6%)	18 (21.7%)	77 (18.0%)
Immunosuppression	115 (66.1%)	127 (74.3%)	61 (73.5%)	303 (70.8%)
Smoking	49 (28.2%)	100 (58.5%)	49 (59.0%)	198 (46.3%)
Anal lesions	18 (10.3%)	17 (9.9%)	7 (8.4%)	42 (9.8%)
**Who is HPV vaccination recommended?**				
Anyone, regardless of age	142 (81.6%)	153 (89.5%)	78 (94.0%)	373 (87.1%)
People from 9 years of age	14 (8.0%)	6 (3.5%)	6 (7.2%)	26 (6.1%)
Only for adults	3 (1.7%)	0 (0.0%)	1 (1.2%)	4 (0.9%)
Only people who have not yet initiated sexual activity	20 (11.5%)	21 (12.3%)	4 (4.8%)	45 (10.5%)
Women only	3 (1.7%)	1 (0.6%)	0 (0.0%)	4 (0.9%)
Do not know	3 (1.7%)	0 (0.0%)	0 (0.0%)	3 (0.7%)

Data are presented as *n* (%). Percentages were calculated using the total number of respondents in each field of study. * Multiple responses were permitted; therefore, percentages do not sum to 100% for this item.

**Table 3:** HPV vaccination status, circumstances and attitudes towards HPV vaccination.

Variable	Public Health (*n* = 174)	Dietetics (*n* = 171)	Physiotherapy (*n* = 83)	Total (*n* = 428)
**HPV vaccination status**				
Completed vaccination schedule	41 (23.6%)	34 (19.9%)	22 (26.5%)	97 (22.7%)
In the process of vaccination	21 (12.1%)	15 (8.8%)	6 (7.2%)	42 (9.8%)
Not vaccinated, but willing to vaccinate	86 (49.4%)	109 (63.7%)	51 (61.4%)	246 (57.5%)
Not vaccinated and unwilling to vaccinate	26 (14.9%)	13 (7.6%)	4 (4.8%)	43 (10.0%)
**Source of recommendation for HPV**				
Primary care physician	5 (2.9%)	4 (2.3%)	2 (2.4%)	11 (2.6%)
Specialist physician	2 (1.1%)	3 (1.8%)	0 (0.0%)	5 (1.2%)
Academic teacher during university lectures	56 (32.2%)	82 (48.0%)	9 (10.8%)	147 (34.3%)
Parents	24 (13.8%)	29 (17.0%)	20 (24.1%)	73 (17.1%)
Friends	2 (1.1%)	0 (0.0%)	5 (6.0%)	7 (1.6%)
Nobody	85 (48.9%)	53 (31.0%)	47 (56.6%)	185 (43.2%)
**Financial burden of HPV vaccination**				
Not a problem	78 (44.8%)	84 (49.1%)	55 (66.3%)	217 (50.7%)
A problem, but vaccination is/was still considered worthwhile	70 (40.2%)	52 (30.4%)	21 (25.3%)	143 (33.4%)
A problem; vaccination was abandoned	15 (8.6%)	31 (18.1%)	5 (6.0%)	51 (11.9%)
Cost irrelevant; would not vaccinate anyway	11 (6.3%)	4 (2.3%)	2 (2.4%)	17 (4.0%)
Main reasons for HPV vaccination *				
Prevention of cervical, vaginal or vulvar cancer	71 (40.8%)	60 (35.1%)	32 (38.6%)	163 (38.1%)
Prevention of penile cancer	45 (25.9%)	40 (23.4%)	5 (6.0%)	90 (21.0%)
Prevention of anal cancer	42 (24.1%)	28 (16.4%)	5 (6.0%)	75 (17.5%)
Prevention of oral/oropharyngeal cancer	40 (23.0%)	24 (14.0%)	9 (10.8%)	73 (17.1%)
Protection of sexual partner from HPV infection	115 (66.1%)	128 (74.9%)	66 (79.5%)	309 (72.2%)
Protection of own health	135 (77.6%)	140 (81.9%)	70 (84.3%)	345 (80.6%)

Data are presented as *n* (%). Percentages were calculated within each field of study. * Multiple responses were permitted; therefore, percentages do not sum to 100% for this item.

**Table 4:** Awareness of and attitudes towards the national publicly funded HPV vaccination programme.

Variable	Public Health (*n* = 174)	Dietetics (*n* = 171)	Physiotherapy (*n* = 83)	Total (*n* = 428)
**Importance of HPV vaccination for public health**				
Yes	151 (86.8%)	158 (92.4%)	76 (91.6%)	385 (90.0%)
No	0 (0.0%)	1 (0.6%)	0 (0.0%)	1 (0.2%)
No opinion	23 (13.2%)	12 (7.0%)	7 (8.4%)	42 (9.8%)
**Awareness of the** **national publicly funded HPV vaccination programme**				
Yes	152 (87.4%)	167 (97.7%)	71 (85.5%)	390 (91.1%)
No	22 (12.6%)	4 (2.3%)	12 (14.5%)	38 (8.9%)
**Eligibility for free HPV vaccination under the programme**				
Girls aged 9–14 years only	32 (18.4%)	26 (15.2%)	14 (16.9%)	72 (16.8%)
Girls and boys aged 9–14 years	116 (66.7%)	138 (80.7%)	61 (73.5%)	315 (73.6%)
Anyone who wishes to be vaccinated	11 (6.3%)	1 (0.6%)	1 (1.2%)	13 (3.0%)
Do not know	15 (8.6%)	6 (3.5%)	7 (8.4%)	28 (6.5%)
**Perceived** **sufficiency of HPV vaccination programme promotion**				
Yes	105 (60.3%)	113 (66.1%)	41 (49.4%)	259 (60.5%)
No	69 (39.7%)	58 (33.9%)	42 (50.6%)	169 (39.5%)
**Involvement in HPV vaccination promotion within the school community**				
Yes	145 (83.3%)	146 (85.4%)	57 (68.7%)	348 (81.3%)
No	29 (16.7%)	25 (14.6%)	26 (31.3%)	80 (18.7%)
**Willingness to deliver HPV education in schools**				
Yes	97 (55.7%)	115 (67.3%)	45 (54.2%)	257 (60.0%)
No	77 (44.3%)	56 (32.7%)	38 (45.8%)	171 (40.0%)

Data are presented as *n* (%). Percentages were calculated within each field of study.

**Table 5:** Previous experiences with recommended (non-mandatory) vaccinations.

Variable	Public Health (*n* = 174)	Dietetics (*n* = 171)	Physiotherapy (*n* = 83)	Total (*n* = 428)
**Number of COVID-19 vaccine doses received**				
None	21 (12.1%)	23 (13.5%)	11 (13.3%)	55 (12.9%)
One dose	38 (21.8%)	50 (29.2%)	18 (21.7%)	106 (24.8%)
Two doses	52 (29.9%)	48 (28.1%)	23 (27.7%)	123 (28.7%)
Three doses	59 (33.9%)	46 (26.9%)	30 (36.1%)	135 (31.5%)
More than three doses	4 (2.3%)	4 (2.3%)	1 (1.2%)	9 (2.1%)
**Fear before the first COVID-19 vaccination dose ^1^**				
Yes	51 (33.3%)	58 (39.2%)	28 (38.9%)	137 (36.7%)
No	102 (66.7%)	90 (60.8%)	44 (61.1%)	236 (63.3%)
**Reasons for fear of COVID-19 vaccination ^2^**				
Fear of adverse effects	34 (66.7%)	36 (62.1%)	16 (57.1%)	86 (62.8%)
Lack of trust in COVID-19 vaccines	15 (29.4%)	19 (32.8%)	11 (39.3%)	45 (32.8%)
Lack of trust in vaccines in general	0 (0.0%)	0 (0.0%)	0 (0.0%)	0 (0.0%)
Lack of access to information about COVID-19 vaccines	0 (0.0%)	1 (1.7%)	0 (0.0%)	1 (0.7%)
Fear of needles	1 (2.0%)	2 (3.4%)	1 (3.6%)	4 (2.9%)
Other	1 (2.0%)	0 (0.0%)	0 (0.0%)	1 (0.7%)
**Influenza vaccination within the previous 3 years**				
Yes	8 (4.6%)	0 (0.0%)	5 (6.0%)	13 (3.0%)
No	166 (95.4%)	171 (100.0%)	78 (94.0%)	415 (97.0%)
**Fear before the first influenza vaccination ^3^**				
Yes	0 (0.0%)	NA	1 (20.0%)	1 (7.7%)
No	8 (100.0%)	NA	4 (80.0%)	12 (92.3%)
**Reasons for fear of influenza vaccination ^4^**				
Fear of adverse effects	0 (0.0%)	NA	1 (100.0%)	1 (100.0%)
Lack of trust in influenza vaccines	0 (0.0%)	NA	0 (0.0%)	0 (0.0%)
Lack of trust in vaccines in general	0 (0.0%)	NA	0 (0.0%)	0 (0.0%)
Lack of access to information about influenza vaccines	0 (0.0%)	NA	0 (0.0%)	0 (0.0%)
Fear of needles	0 (0.0%)	NA	0 (0.0%)	0 (0.0%)
Other	0 (0.0%)	NA	0 (0.0%)	0 (0.0%)

Data are presented as *n* (%). Percentages for vaccination status were calculated using the total number of respondents in each field of study. ^1^ Asked only of respondents who had received at least one dose of the COVID-19 vaccine; denominator: *n* = 153, 148, and 72, respectively. ^2^ Asked only of respondents who reported fear before their first COVID-19 vaccination; denominator: *n* = 51, 58, and 28, respectively. ^3^ Asked only of respondents who had received at least one influenza vaccine dose; denominator: *n* = 8, 0, and 5, respectively. ^4^ Asked only of respondents who reported fear before their first influenza vaccination; denominator: *n* = 0, 0, and 1, respectively. NA, not applicable.

**Table 6:** General attitudes towards vaccination.

Statement	Public Health (*n* = 174)	Dietetics (*n* = 171)	Physiotherapy (*n* = 83)	Total (*n* = 428)
**General support for vaccination**				
Strongly agree	92 (52.9%)	129 (75.4%)	71 (85.5%)	292 (68.2%)
Agree	61 (35.1%)	34 (19.9%)	9 (10.8%)	104 (24.3%)
No opinion	21 (12.1%)	8 (4.7%)	3 (3.6%)	32 (7.5%)
Disagree	0 (0.0%)	0 (0.0%)	0 (0.0%)	0 (0.0%)
Strongly disagree	0 (0.0%)	0 (0.0%)	0 (0.0%)	0 (0.0%)
**Support for childhood vaccination**				
Strongly agree	102 (58.6%)	137 (80.1%)	75 (90.4%)	314 (73.4%)
Agree	58 (33.3%)	26 (15.2%)	6 (7.2%)	90 (21.0%)
No opinion	14 (8.0%)	8 (4.7%)	2 (2.4%)	24 (5.6%)
Disagree	0 (0.0%)	0 (0.0%)	0 (0.0%)	0 (0.0%)
Strongly disagree	0 (0.0%)	0 (0.0%)	0 (0.0%)	0 (0.0%)
**Vaccination during pregnancy**				
Strongly agree	49 (28.2%)	62 (36.3%)	40 (48.2%)	151 (35.3%)
Agree	54 (31.0%)	47 (27.5%)	19 (22.9%)	120 (28.0%)
No opinion	58 (33.3%)	55 (32.2%)	23 (27.7%)	136 (31.8%)
Disagree	10 (5.7%)	6 (3.5%)	0 (0.0%)	16 (3.7%)
Strongly disagree	3 (1.7%)	1 (0.6%)	1 (1.2%)	5 (1.2%)
**Support for vaccination promotion among healthcare professionals**				
Strongly agree	92 (52.9%)	105 (61.4%)	54 (65.1%)	251 (58.6%)
Agree	53 (30.5%)	43 (25.1%)	9 (10.8%)	105 (24.5%)
No opinion	25 (14.4%)	22 (12.9%)	20 (24.1%)	67 (15.7%)
Disagree	3 (1.7%)	1 (0.6%)	0 (0.0%)	4 (0.9%)
Strongly disagree	1 (0.6%)	0 (0.0%)	0 (0.0%)	1 (0.2%)
**Interest in vaccination before travel to exotic destinations**				
Strongly agree	111 (63.8%)	134 (78.4%)	74 (89.2%)	319 (74.5%)
Agree	49 (28.2%)	29 (17.0%)	6 (7.2%)	84 (19.6%)
No opinion	13 (7.5%)	8 (4.7%)	2 (2.4%)	23 (5.4%)
Disagree	1 (0.6%)	0 (0.0%)	1 (1.2%)	2 (0.5%)
Strongly disagree	0 (0.0%)	0 (0.0%)	0 (0.0%)	0 (0.0%)

Data are presented as *n* (%). Percentages were calculated within each field of study.

## Data Availability

The dataset we analysed for the current study is available from the corresponding author on request.

## Supplementary Materials

The following supporting information can be downloaded at https://www.mdpi.com/article/10.3390/vaccines14100907/s1, Figure S1: The survey questionnaire. Table S1: Associations between field of study and vaccination-related characteristics. Table S2: Associations between gender and vaccination status.
