# Supplementary material for: HPV Vaccination Among Students of Health-Related Disciplines: Knowledge, Intentions, Attitudes, and Barriers
Source: Vaccines (Basel). 2026 Oct 18;14(10):907. doi: 10.3390/vaccines14100907 (PMC13709627; doi:10.3390/vaccines14100907)
Supplement: Supplementary file 1 [file vaccines-14-00907-s001.zip › vaccines-4596270-supplementary.pdf]

Fig. S1. The survey questionnaire.

Knowledge, Attitudes and Practices Regarding HPV Vaccination

The survey is completely anonymous. Participation is voluntary, and you may discontinue completing the questionnaire at any time without any consequences. Submission of the completed questionnaire constitutes informed consent to participate in the study. The results of the survey will be used for scientific research purposes. Thank you in advance for taking the time to complete the survey.

Part I. Sociodemographic characteristics

1. Field of study
- o Public Health
- o Dietetics
- o Physiotherapy

2. Level of study
- o Bachelor's degree
- o Master's degree

3. Sex
- o Female
- o Male

4. Age

....

5. Place of origin
- o City with >100,000 inhabitants
- o City with 20,000–100,000 inhabitants
- o City with <20,000 inhabitants
- o Village

6. Have you initiated sexual activity?
- o Yes
- o No

7. Have you visited a gynecologist/urologist within the last 2 years?
- o Yes
- o No

Part II. HPV vaccination status and attitudes.

8. What is your current HPV vaccination status?
- o Vaccinated
- o Currently undergoing vaccination
- o Not vaccinated, but I would like to be vaccinated
- o Not vaccinated and I do not intend to be vaccinated

9. Who recommended that you get vaccinated against HPV?

For respondents who had received HPV vaccine.
- o General practitioner / primary care physician
- o Specialist physician
- o Academic teacher/lecturer during classes at the university
- o Parents
- o Friend/classmate
- o Nobody, I looked for it myself.

10. HPV vaccines for adults are partially or fully paid for. Would the cost of vaccination be a problem for you?

- No, the cost would not be a problem
- Yes, the cost would be a problem, but I have been vaccinated / I still believe that I should be vaccinated
- Yes, the cost would be a problem and I had to give up vaccination
- It does not matter whether the vaccine is paid or free; I would not get vaccinated anyway

11. What do you consider the most important reasons for HPV vaccination? *Select all answers that you consider important.*

- Protection against cervical, vaginal, or vulvar cancer
- Protection against penile cancer
- Protection against anal cancer
- Protection against oral cavity and throat cancer
- Protection of one's sexual partner against HPV infection
- Protection of own health.

Part III. Basic knowledge about HPV and HPV vaccination

12. Expand the abbreviation HPV.

…………………………………………………………………………………….

13. What is the most common route of HPV transmission?

- Respiratory route
- Foodborne route
- Sexual contact
- Skin contact

14. Which of the following are risk factors for HPV infection in women? *Select all answers that you consider appropriate.*

- Early initiation of sexual activity
- Multiple sexual partners
- Reduced immunity / immunosuppression
- Cigarette smoking
- Infections such as chlamydia

15. Which of the following are risk factors for HPV infection in men? *Select all answers that you consider appropriate.*

- Multiple sexual partners
- Chronic inflammation of the urethra and glans
- Reduced immunity / immunosuppression
- Cigarette smoking
- Lesions or pathological changes in the anal area

16. Who should be vaccinated against HPV recommend?

- Anyone, regardless of age
- People from 9 years of age
- Only for adults
- Only people who have not yet initiated sexual activity
- Women only
- I do not know

Part IV. Awareness of and attitudes towards the HPV vaccination programme in Poland

17. Do you think that HPV vaccination is important for public health?

- Yes
- No
- I have no opinion

18. Have you heard about the national publicly funded HPV vaccination programme?

- Yes
- No

19. Who is eligible for free HPV vaccination under the programme?

- Girls aged 9–14 years only
- Girls and boys aged 9–14 years
- Anyone who wishes to be vaccinated
- I do not know

20. Do you think that the Polish HPV vaccination programme is sufficiently promoted?

- Yes
- No

21. Do you think final-year students of health-related disciplines should be involved in promoting HPV vaccination within the school community?

- Yes
- No

22. If you were asked to give an educational talk at a school about HPV infection and HPV vaccination, would you be willing to do so?

- Yes
- No

Part V. Previous experience with other recommend (non-mandatory) vaccination

COVID-19 vaccination

23. How many doses of COVID-19 vaccine have you received?

- None
- One dose
- Two doses
- Three doses
- More than three doses

24. Did you experience anxiety or fear before your first COVID-19 vaccination? *For respondents who had received at least one COVID-19 vaccine dose.*

- Yes
- No

25. What caused your concerns about receiving the COVID-19 vaccine? *For respondents who reported fear or anxiety before vaccination.*

- Concern about possible adverse effects following vaccination
- Lack of trust in the COVID-19 vaccine
- Lack of trust in vaccines in general
- Lack of access to information about the COVID-19 vaccine
- Fear of needles
- Other

Influenza vaccination

26. Have you been vaccinated against influenza within the last 3 years?

- Yes
- No

27. Did you experience anxiety or fear before your first influenza vaccination?
For respondents who had received an influenza vaccine.

- o Yes
- o No

28. What caused your concerns about receiving the influenza vaccine?
For respondents who reported fear or anxiety before vaccination.

- o Concern about possible adverse effects following vaccination
- o Lack of trust in the influenza vaccine
- o Lack of trust in vaccines in general
- o Lack of access to information about the influenza vaccine
- o Fear of needles
- o Other

Part VI. General attitudes towards vaccination

For the following statements, please indicate your level of agreement.

29. I support vaccination in general.

- o Strongly agree
- o Agree
- o Neither agree nor disagree / No opinion
- o Disagree
- o Strongly disagree

30. I support vaccination of children.

- o Strongly agree
- o Agree
- o Neither agree nor disagree / No opinion
- o Disagree
- o Strongly disagree

31. I would recommend vaccination during pregnancy.

- o Strongly agree
- o Agree
- o Neither agree nor disagree / No opinion
- o Disagree
- o Strongly disagree

32. I would promote recommended (non-mandatory) vaccinations among healthcare professionals.

- o Strongly agree
- o Agree
- o Neither agree nor disagree / No opinion
- o Disagree
- o Strongly disagree

33. Interest in recommended vaccinations before travelling to an exotic destination, for example in Asia or Africa.

- o Strongly agree
- o Agree
- o Neither agree nor disagree / No opinion
- o Disagree
- o Strongly disagree .

Table S1. Associations between field of study and vaccination-related characteristics.

Characteristic	Statistical test	$\chi^2(2)$	p	Significant pairwise comparisons after Holm adjustment	Effect size
HPV vaccination status	Pearson's χ^2	1.989	0.370	—	
COVID-19 vaccination status	Pearson's χ^2	0.162	0.922	—	
Influenza vaccination status	Fisher–Freeman–Halton exact test	—	0.003	Dietetics vs Public Health ($p = 0.014$)<br>Dietetics vs Physiotherapy ($p = 0.010$)	*<br>*
Awareness of the national publicly funded HPV vaccination programme	Pearson's χ^2	15.281	<0.001	Dietetics vs Public Health (p <0.001);<br>Dietetics vs Physiotherapy (p <0.001)	OR = 6.06 (95% CI: 2.04–17.86)<br>OR = 7.06 (95% CI: 2.20–22.63)
Knowledge of programme eligibility	Pearson's χ^2	8.743	0.013	Dietetics vs Public Health ($p = 0.009$)	OR = 2.09 (95% CI: 1.28–3.43)
Support for HPV vaccination promotion in school community	Pearson's χ^2	11.052	0.004	Public Health vs Physiotherapy ($p = 0.014$)<br>Dietetics vs Physiotherapy ($p = 0.005$)	OR = 2.28 (95% CI: 1.24–4.20)<br>OR = 2.66 (95% CI: 1.42–4.99)
Willingness to deliver HPV education in schools	Pearson's χ^2	6.217	0.045	—	
General support for vaccination	Pearson's χ^2	9.030	0.011	Dietetics vs Public Health ($p = 0.040$)	OR = 2.80 (95% CI: 1.20–6.50)
Support for childhood vaccination	Pearson's χ^2	3.951	0.139	—	
Support for vaccination of pregnant women	Pearson's χ^2	3.442	0.179	—	
Support for vaccination promotion among healthcare workers	Pearson's χ^2	4.531	0.104	—	
Pre-travel vaccination for exotic destinations	Pearson's χ^2	2.707	0.258	—	

Data were analyzed according to the dichotomized response categories defined a priori for statistical analysis. HPV vaccination status was categorized as having initiated vaccination (≥1 dose) vs. not vaccinated. COVID-19 vaccination status was categorized as vaccinated (≥1 dose) vs. not vaccinated. Knowledge of HPV programme

eligibility was categorized as correct (girls and boys aged 9–14 years) vs. incorrect/don't know. For attitudinal variables, “strongly agree” and “agree” were combined as yes, whereas “no opinion”, “disagree”, and “strongly disagree” were combined as no opinion/no. Pearson's χ^2 test was used for 2 × 3 contingency tables when its assumptions were met. For influenza vaccination, the Fisher–Freeman–Halton exact test was used because of small expected frequencies. For significant omnibus tests, pairwise comparisons were performed with Holm adjustment for multiple comparisons. OR, odd ratio; CI, confidence interval. * - Due to the small number of events and zero cell counts, OR estimates are unstable and are therefore not reported.

Table S2. Associations between gender and vaccination status.

Vaccination status	Statistical test	$\chi^2(1)$	p (women vs. men)	Effect size
HPV vaccination	Pearson's χ^2	18.739	<0.001	OR = 2.77 (95% CI: 1.73–4.44)
COVID-19 vaccination	Pearson's χ^2	0.180	0.671	—
Influenza vaccination	Fisher's exact test	—	0.557	—

HPV vaccination status was categorized as having initiated vaccination (≥1 dose) vs. not vaccinated. COVID-19 vaccination status was categorized as vaccinated (≥1 dose) vs. not vaccinated. Fisher's exact test was used for influenza vaccination because of low frequencies. OR, odd ratio; CI, confidence interval.
